# Morphology, phylogeny, and ecology of the aphelids (Aphelidea, Opisthokonta) and proposal for the new superphylum Opisthosporidia

**DOI:** 10.3389/fmicb.2014.00112

**Published:** 2014-03-28

**Authors:** Sergey A. Karpov, Maria A. Mamkaeva, Vladimir V. Aleoshin, Elena Nassonova, Osu Lilje, Frank H. Gleason

**Affiliations:** ^1^Zoological Institute, Russian Academy of SciencesSt. Petersburg, Russian Federation; ^2^St. Petersburg State UniversitySt. Petersburg, Russian Federation; ^3^A. N. Belozersky Institute for Physico-Chemical Biology, Lononosov Moscow State UniversityMoscow, Russian Federation; ^4^Institute of Cytology, Russian Academy of SciencesSt. Petersburg, Russian Federation; ^5^School of Biological Sciences F07, University of SydneySydney, NSW, Australia

**Keywords:** Opisthosporidia, Aphelida, Cryptomycota, Microsporidia, *Rozella*, ultrustucture, molecular phylogeny, ecology

## Abstract

The aphelids are a small group of intracellular parasitoids of common species of eukaryotic phytoplankton with three known genera *Aphelidium, Amoeboaphelidium,* and *Pseudaphelidium,* and 10 valid species, which form along with related environmental sequences a very diversified group. The phyla Microsporidia and Cryptomycota, and the class Aphelidea have recently been considered to be a deep branch of the Holomycota lineage forming the so called the ARM-clade which is sister to the fungi. In this review we reorganize the taxonomy of ARM-clade, and establish a new superphylum the Opisthosporidia with three phyla: Aphelida phyl. nov., Cryptomycota and Microsporidia. We discuss here all aspects of aphelid investigations: history of our knowledge, life cycle peculiarities, the morphology (including the ultrastructure), molecular phylogeny, ecology, and provide a taxonomic revision of the phylum supplied with a list of species. We compare the aphelids with their nearest relatives, the species of *Rozella*, and improve the diagnosis of the phylum Cryptomycota.

## INTRODUCTION

The aphelids are a small group of intracellular parasitoids of algae, which are currently placed in the class Aphelidea (Gromov, 2000). The class Aphelidea includes two freshwater genera, *Aphelidium* and* Amoeboaphelidium,* and a marine genus, *Pseudaphelidium.* Although for a long time members of the class Aphelidea had uncertain affinities, the phylogenetic position of this class has been recently clarified by molecular phylogenetic analyses of *Amoeboaphelidium protococcarum* (Karpov et al., 2013). The aphelids belong to the supergroup Opisthokonta, which includes multicellular animals and fungi, and a variety of unicellular organisms, which over the past decade the molecular phylogeny has been tied to each of the two following major clades (Paps et al., 2013). The Metazoa, Choanoflagellata, and Mesomycetozoea now form the Holozoa (Torruella et al., 2012), whereas nucleariid amoebae, fungi, rozellids (Cryptomycota), aphelids, and microsporidia form the Holomycota (Liu et al., 2009; Lara et al., 2010; Jones et al., 2011a; Karpov et al., 2013; Letcher et al., 2013). Recent molecular phylogeny analyses show that the class Aphelidea is sister to both Microsporidia and Cryptomycota (Karpov et al., 2013). This strongly argues in favor of the re-classification of Aphelidea at the phylum rank. All the three phyla form a separate branch sister to classical (“true”) fungi, which include Dikarya (Ascomycota and Basidiomycota), paraphyletic Zygomycota, and Chytridiomycota *sensu lato* (Voigt et al., 2013).

This review focuses on the aphelids and discusses their phylogeny, life cycle, morphology, ecology, and their taxonomy. Because of their close phylogenetic relationship and life cycle similarity (in contrast with the fast-evolving Microsporidia), we often consider them in comparison with Cryptomycota. Indeed, the aphelids have a life cycle similar to that of the Cryptomycota, but are parasitoids of algae, and not of zoosporic fungi and Oomycetes as are the known species of *Rozella*.

## APHELID RELATIONSHIPS: HISTORICAL SKETCH

Historical interpretations of the phylogenetic affinities of the aphelids were thoroughly discussed by Gromov (2000). Therefore, we only highlight here some of the important points. The research on aphelids began in the 19th century when the genus *Aphelidium* Zopf was first described (Zopf, 1885). 40 years later in 1925, *Amoeboaphelidium* Scherffel was described, and both these organisms were treated as the Cienkowski’s “Monadinea” group, comprised of the extremely divergent “fungal animals” – organisms with a fungal-like life cycle, but having an amoeboid trophic stage (ref. in Gromov, 2000). In the 1950–1960s, the aphelids were included in the order Proteomyxida or subclass Proteomyxidia within the class Rhizopoda (Hall, 1953; Honigberg et al., 1964; Kudo, 1966). However, subsequently these protists were completely forgotten in classifications in later years (Levine et al., 1980; Karpov, 1990; Page and Siemensma, 1991; Cavalier-Smith, 1993, 1996/1997). This is difficult to understand because during the 1960s and 1970s a number of articles were written on *Aphelidium* and *Amoeboaphelidium* by Schnepf et al. (1971) and Gromov et al. (ref. in Gromov, 2000). By the end of the last century we knew much more about the life cycles, ultrastructure and biological peculiarities of several species of aphelids. Gromov (2000) reviewed this material and established a new class Aphelidea for *Aphelidium* Zopf, 1885, *Amoeboaphelidium* Scherffel, 1925, and *Pseudaphelidium* Schweikert et Schnepf, 1996.

Until recently, the relationship between the aphelids and fungi was unclear. Cavalier-Smith (1998) suggested that the genus *Aphelidium* belongs to the opisthokonts because of their posteriorly directed uniflagellate zoospores and flat mitochondrial cristae. Gromov (2000) placed the class Aphelidea in the phylum Rhizopoda *sensu lato* on the basis of the amoeboid nature of the trophozoite stage, despite an unpublished 18S rRNA partial sequence of *Amoeboaphelidium protococcarum* which suggested a relationship with Choanozoa (Pinevich et al., 1997). Later Karpov (Adl et al., 2005) transferred the class Aphelidea into the phylum Mezomycetozoea based on the preliminary 18S rRNA molecular phylogeny of* Aphelidium*. In addition to these classifications based on the 18S rRNA marker, aphelids were placed within Ichthyosporea based on their parasitic nature (Shalchian-Tabrizi et al., 2008), and then, based on their morphology and lifestyle, as a “new” order Aphelidida, class Rozellidea, in the new subphylum Paramycia Cavalier-Smith (2013) of the phylum Choanozoa Cavalier-Smith, 1981 (Cavalier-Smith, 2013). The creation of a “new” Aphelidida order was unjustified, since that order had already been established by Gromov (2000) earlier.

**FIGURE 1:**
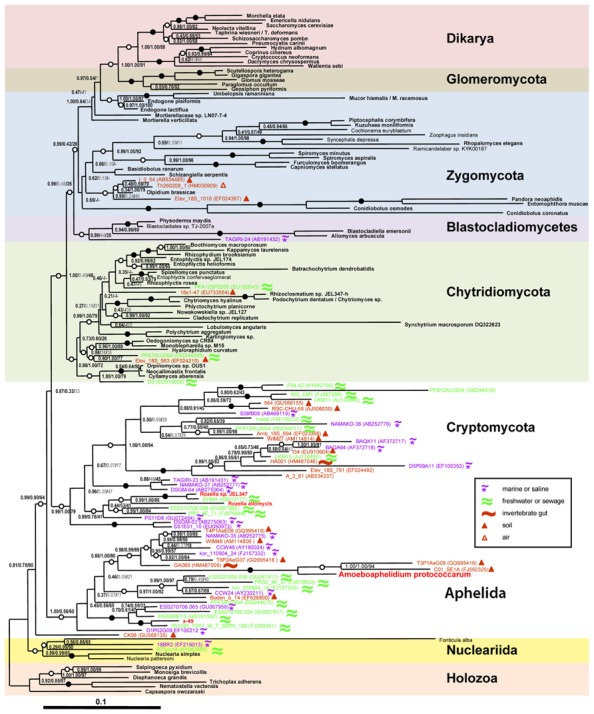
**Position of *Amoeboaphelidium protococcarum* on the tree inferred from rDNA analyses by Bayesian and ML methods (after: Karpov et al., 2013)**. PhyloBayes tree topology was calculated from an alignment of 144 sequences and 4,384 nucleotide characters. Node support values are given as follows: Bayesian posterior probabilities (PhyloBayes/MrBayes) followed by bootstrap values (RAxML). We used the GTR + CAT model without partition by genes for PhyloBayes calculations and the same model with partition by genes for RAxML calculations. The GTR + I + Γ_12_ model was used for MrBayes calculations. Filled circles indicate that all support values are above 98%; empty circles indicate that at least one support value is above 98%. Shown in gray are support values for clades not included in the consensus trees. Symbols in the inset indicate habitats for environmental sequences. Scale bar indicates substitutions per site.

Previous classifications attempts based on 18S rRNA partial gene sequences were affected by the limited resolution of this marker for the eukaryotic tree. Only recently the molecular phylogeny of *Amoeboaphelidium protococcarum* (strain x-5 CALU), based on five genes (RPB1, RPB2, 18S, 28S, and 5.8S rRNA), unambiguously showed that the aphelids branch together with Cryptomycota (*Rozella* + related environmental sequences) and microsporidia forming the ARM (Aphelidea + *Rozella* + Microsporidia) branch (Karpov et al., 2013; **Figure 1**). Letcher et al. (2013) confirmed the phylogenetic position of *Amoeboaphelidium* by isolating one more strain (FD01) of *Amoeboaphelidium protococcarum* and studying its ultrastructure and molecular phylogeny based on 18S, 5.8S, and 28S rRNA gene sequences.

The fact that the aphelids form a monophyletic group with cryptomycota/rozellids and microsporidia to the exclusion of Chytridiomycota and other fungi is also reflected in the possession of a distinctive morphological feature. According to Gromov (2000) a unique characteristic of aphelids is the intracellular trophic stage which is amoeboid and which engulfs the host cell contents. A similar stage is found in *Rozella* (Powell, 1984), but is absent in fast evolving and highly derived Microsporidia (Vávra and Lukeš, 2013). This characteristic strongly differentiates the aphelids and *Rozella* from Chytridiomycota and other fungi, and is unambiguously supported by molecular phylogeny of *Rozella allomycis* and two strains of *Amoeboaphelidium protococcarum* together with their related environmental sequences (James et al., 2013; Karpov et al., 2013; Letcher et al., 2013).

## LIFE CYCLES

**FIGURE 2:**
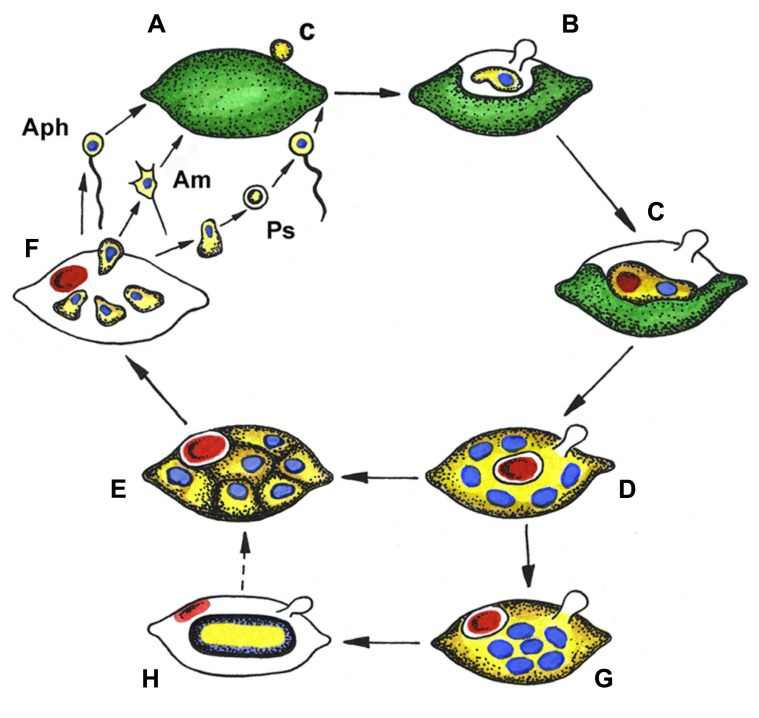
**Generalized life cycle of aphelids.** *Aphelidium* (Aph), *Amoeboaphelidium* (Am) and *Pseudaphelidium* (Ps), distinguished by zoospore structure and development. **(A)** Zoospore encystment (c) on the host surface, **(B)** propagule penetration into the host, **(C)** trophic amoeba with nucleus (blue) and residual body (red) engulfs host cytoplasm, **(D)** multinuclear plasmodium (yellow) totally replaced the host, contains several nuclei (blue) and central vacuole with residual body (red), **(E)** plasmodium divides producing uninuclear cells, **(F)** mature zoospores released from the empty host cell, **(G)** precursory stage to the resting spore with nuclei in the center, **(H)** resting spore with ejected residual body. Dotted line shows conceivable way from resting spore to divided plasmodium. Colors: green, host (alga) cytoplasm; yellow, parasitoid cytoplasm; blue, nucleus; red, residual body.

The complex life cycles of *Aphelidium, Amoeboaphelidium,* and* Pseudaphelidium* appear to be very similar to each other (**Figure 2**) and superficially similar to those of many species of Chytridiomycota with endobiotic development in their algal host cells (Gromov, 2000).

**FIGURE 3:**
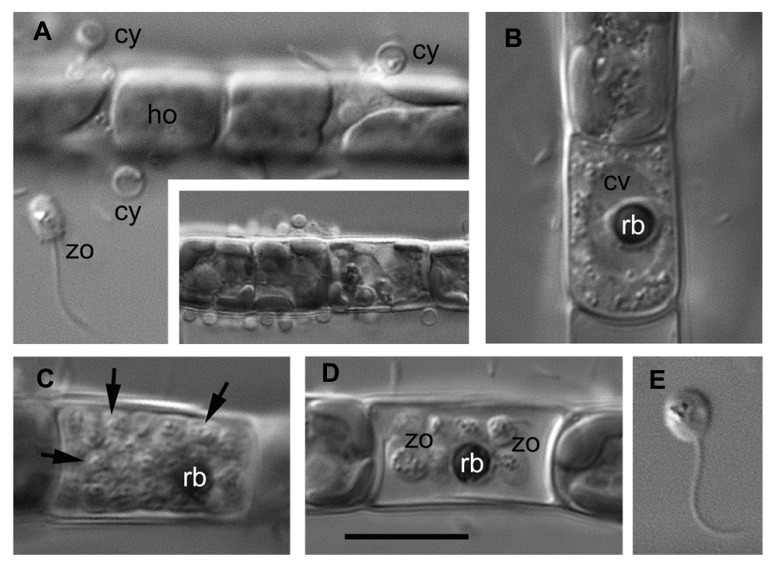
**Main stages of the life cycle of *Aphelidium* sp. parasitizing on *Tribonema gayanum* CALU- 20.** Living cells observed under DIC. **(A)** Zoospore (zo) before attachment to the host (ho) and cysts (cy) on the surface of *Tribonema* filament. Insert – multiple infection at lower magnification. **(B)** Plasmodium with central vacuole (cv) and residual body (rb). **(C)** multicellular stage of parasitoid with rb at the periphery (arrows show separate cells). **(D)** mature zoospores with flagella and residual body in the empty host cell. **(E)** Free-swimming zoospore at high magnification. Scale bar: **A–D**, 10 μm, insertion in **A**, 15 μm, **E**, 8 μm.

As an example, the *Aphelidium* opisthokont zoospore attaches to the host algae and encysts while losing its flagellum (**Figure 3A**). A cyst germinates and penetrates the host cell wall with an infection tube. The posterior vacuole appears in the cyst, enlarges, and then pushes the contents of the cyst into the interior of the host cell through the infection tube (**Figures 2** and **3A**). The parasitoid becomes the intracellular phagotrophic amoeba which engulfs the host cytoplasm with pseudopodia and transports the food into a central digestive vacuole. The parasitoid grows forming an endobiotic plasmodium with residual body while it totally consumes the cytoplasm of the host cell (**Figures 2** and **3B**). A multinucleate plasmodium is formed with a large central vacuole and a residual excretory body. The parasitoid does not form its own sporangium wall; rather it uses the host cell walls as the sporangium wall (**Figure 3C**). The mature plasmodium then divides into a number of uninucleated cells (**Figures 2** and **3C,D**). After maturation, the uniflagellated zoospores of *Aphelidium* are released from the empty host cell through the hole made previously by the infection tube and infect other algae (**Figures 2** and **3D,E**).

All known aphelids can produce multiple infections (**Figures 2** and **3**). In the case of *Amoeboaphelidium,* several amoebae grow separately in the infected alga but later fuse into a multinucleate cell, forming a single plasmodium in each infected cell (Gromov and Mamkaeva, 1968).

Sometimes giant multinucleate amoebae are released along with the uninucleate amoebae from the sporangium (Gromov and Mamkaeva, 1968). The origin of these giant amoebae is not clear, but they might result from the incomplete divisions of the mature plasmodium (Gromov and Mamkaeva, 1968). Most of these giants died after a short period of activity in culture.

**FIGURE 4:**
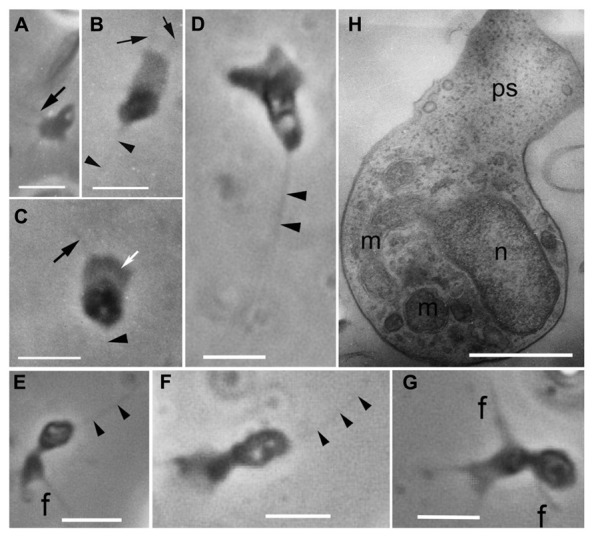
**Amoeboid zoospores of *Amoeboaphelidium protococcarum.*** Living cells observed using phase contrast. **(A–C)** Strain x-1 CALU, **(D–G)** strain x-5 CALU. **(H)** Amoeba of *Amoeboaphelidium protococcarum* strain x-5 CALU on ultrathin longitudinal section. **(D–H)** After: Karpov et al. (2013). f and black arrows, filopodia often producing by broad anterior pseudopodium (ps, white arrow), m, mitochondrium, n, nucleus, arrowheads show pseudocilium. Scale bars: **A–C**, 3 μm; **D–G**, 2 μm; **H**, 1 μm.

Originally, amoeboid zoospores without any traces of flagella were described for *Amoeboaphelidium* (Scherffel, 1925; Gromov and Mamkaeva, 1968; Gromov, 2000). Further investigations of *Amoeboaphelidium protococcarum* (strain x-5 CALU) showed that amoebae actually have a posterior immobile pseudocilium (**Figure 4** – see discussion below). Gromov (2000) assumed that the type of propagule produced is genus specific. Indeed, the *Aphelidium* produces zoospores with a posterior flagellum (**Figures 2** and **3**), *Amoeboaphelidium* produces amoeboid zoospores with posterior pseudocilium (**Figures 2** and **4**), and* Pseudaphelidium* produces amoebae, which encyst. The cysts release one, two or, more often, four uniflagellated zoospores after germination (**Figure 2**). *Pseudaphelidium* zoospores lack the refractive globule, characteristic of *Aphelidium*.

Some aphelid species produce dormant or resting spores which are thought to be resistant to environmental extremes, and/or the result of sexual reproduction (Gromov and Mamkaeva, 1968, 1975; Letcher et al., 2013). In this case, the plasmodium ejects the residual body into the space between the cell wall of the alga and the surface of the parasitoid. The plasmodium then slightly constricts, produces a thick yellowish cell wall inside the empty host and becomes a resting cyst or dormant spore (**Figure 2**). The nuclei of the spore aggregate into the central irregular mass. During germination a spore wall becomes thin and colorless, and the plasmodium divides into several rounded cells. Further proliferation is not observed.

## ULTRASTRUCTURE

### MITOCHONDRION CRISTAE

The first species examined in electron microscope studies were *Amoeboaphelidium chlorellavorum* (Gromov and Mamkaeva, 1970a) and* Amoeboaphelidium protococcarum* (Gromov and Mamkaeva, 1970b). The authors fixed the samples using potassium permanganate, or osmium tetroxide (without glutaraldehyde). The latter fixative gave better results, but mitochondrial cristae appeared rounded in many sections (Gromov and Mamkaeva, 1970a,b). When *Pseudaphelidium drebesii* was fixed with glutaraldehyde the mitochondrial cristae appeared flat in cysts, but looked tubular in trophonts (Schweikert and Schnepf, 1997). These observations gave rise to the characterization of this group as having tubular cristae in the mitochondria (Gromov, 2000). In the recent studies of *Aphelidium,* the mitochondrion had lamellar cristae in zoospores and tubular cristae in cysts (Gromov and Mamkaeva, 1975). The amoebae of *Amoeboaphelidium* also have mitochondria with lamellar cristae (Karpov et al., 2013; Letcher et al., 2013). Altogether, these observations of the mitochondrion ultrastructure suggest that the shape of the mitochondrial cristae is variable in the aphelids.

### FLAGELLATED ZOOSPORES

The flagellated zoospore structure was only described for *Aphelidium* (Gromov and Mamkaeva, 1975). It has one posteriorly directed acronematic flagellum with 9 + 2 axoneme, and few short filopodia at the anterior end, used for cell attachment. The nucleus is located in the anterior part of the cell. A dictyosome lies between the nucleus and the flagellar kinetosome and some mitochondria with lamellate cristae and small lipid droplets are situated around the nucleus. Ribosomes are scattered in the cytoplasm, which usually has few ER cisternae. Unfortunately, the most informative and phylogenetically important flagellar apparatus has not been studied in detail. A short centriole lies at different angles to the kinetosome from nearly parallel (Gromov and Mamkaeva, 1975) to orthogonal (Karpov, in preparation), and both are connected to each other by a rather broad fibrillar bridge. Both the amoeboid and flagellated zoospores of aphelids are uninucleated and have, in addition to the mitochondria, a small microbody with granular contents associated with the nucleus, and several lipid globules spread throughout the cytoplasm. Ribosomal aggregation is absent.

### PLASMODIUM

The ultrastructure of the plasmodium of* Amoeboaphelidium* species does not differ significantly from that of *Aphelidium chlorococcarum* (Gromov and Mamkaeva, 1970a,b), and *Pseudaphelidium* (Schweikert and Schnepf, 1997). The multicellular stage in plasmodium development was shown for *Aphelidium* (Gromov and Mamkaeva, 1975), *Pseudaphelidium* (Schweikert and Schnepf, 1997), and *Amoeboaphelidium* (Letcher et al., 2013).

### PENETRATION APPARATUS

Aphelids have a peculiar penetration apparatus, similar in some respects to that of microsporidia. After zoospore attachment its pseudopodium grows along the host surface seeking a hole or gap in the wall. This was shown for *Aphelidium* (Gromov and Mamkaeva, 1975) and *Pseudaphelidium* (Schweikert and Schnepf, 1997). If the pseudopodium does not find a point of entry into the host, the infection fails. It seems the penetration tube grows around the pseudopodium. After tube penetration in the host, the posterior vacuole enlarges and pushes the parasitoid out of the cyst through the infection tube into the host cell (Gromov, 2000; Karpov et al., 2013). The mechanism of injection needs the cyst wall to generate the pressure inside the cyst for cell migration. In *Pseudaphelidium* the cyst wall is thicker than in other genera, and, unlike other aphelids, a special inverted tube is present in the cyst (Schweikert and Schnepf, 1997). The proximal part of the tube is fixed at a certain place (faced to the host surface) of the cyst similar to that of the anchoring disk complex in the spore of microsporidia. This tube everts during invasion penetrating the host cell wall. In both these respects the tube is more similar to the injection apparatus of microsporidia, than to that of other aphelids. The injection apparatus of microsporidia consists of the injection tube, the polaroplast (membrane storage organelle) and a posterior vacuole (a pressure-building organelle; Vávra and Lukeš, 2013). The precise mechanism of spore extrusion is still unclear (Vávra and Lukeš, 2013).

### AMOEBOID ZOOSPORES

The better studied *Amoeboaphelidium* has two forms of amoebae (aplanospores): so called “radiosa” forms (floating amoebae detached from the substrate), and freely moving amoebae. The floating amoebae are normally spherical and have radial filopodia independently of the shape of the moving morphotype. The moving amoebae are very characteristic of *Amoeboaphelidium protococcarum*: they are subdivided into two nearly equal parts: the posterior which contains the nucleus and other organelles, and the anterior which is made up of the broad and flat pseudopodium producing several thin filopodia (subfilopodia; Karpov et al., 2013; Letcher et al., 2013; **Figure 4H**). The anterior part does not contain any organelles, just a hyaloplasm. The posterior end bears a pseudocilium, which is clearly visible in both the old photographs of x-1 CALU (**Figures 4A–C**), and the recent photographs of x-5 CALU (**Figures 4D–F**). The pseudocilium contains 2–3 microtubules, at least, and seems to represent the rudimentary flagellum (Karpov et al., 2013).

Since the type strain of *Amoeboaphelidium protococcarum* (x-1 CALU) has a pseudocilium, the description for this species has been corrected (see Taxonomy). Although Letcher et al. (2013) paid particular attention to the presence/absence of pseudocilium in FD01, the authors seem to have overlooked this tiny structure, which may have been masked by the very dense cytoplasm. In some of their figures (Figures 5G,H in Letcher et al., 2013), however, the oblique profile of a microtubule is clearly visible. Although the authors marked this microtubule as the filaments, the distance between these “filaments” is precisely 25 nm – the diameter of microtubule. This suggests that the amoeboid zoospore of FD01 also has a pseudocilium.

Zoospores of the strains x-1 and x-5 have a nucleus of peculiar convex-concave shape with the microbody in the invagination. The nucleus in FD01 is more spherical and of convex-flat shape, but is also associated with the microbody. These morphological differences seem not to be significant. The organisms of all three strains (x-1, x-5 CALU, and FD01) are morphologically very similar to each other in the following ways: amoebae have the same dimensions (2–4 μm), moving morphotype, an outer and internal morphology; they infect the green chlorococcous alga *Scenedesmus*; and their life cycle contains all the stages described for *Amoeboaphelidium protococcarum.* Thus, by these criteria they should all certainly belong to the same species, *Amoeboaphelidium protococcarum.*

## MORPHOLOGICAL IDENTITY VS. GENETIC HETEROGENEITY

While morphologically similar, the two strains of *Amoeboaphelidium protococcarum* FD01 from Texas (USA) and x-5 from the Russian Far East exhibited a low similarity in rRNA gene sequences, suggesting a significant phylogenetic distance. Indeed, the similarities were of 86, 84, and 78% for the 18S, the 5.8S, and the 28S rRNA genes respectively (Letcher et al., 2013). For the majority of protists, such differences would correspond to genus level differences at least. For instance, the genus level is placed at 6–10% dissimilarity in 18S rRNA genes for bicosoecids (Kim et al., 2010). It is uncertain whether such genetic distances are normal intraspecific variations for the aphelids, or not. We suggest at this stage of study that both strains should be retained in the genus *Amoeboaphelidium,* but they might actually belong to different species.

Strain diversity in *Amoeboaphelidium protococcarum* was noticed earlier. Pinevich et al. (1997) compared approximate sizes and numbers of chromosomes in strains x-1 and x-5 CALU using pulsed-field gel electrophoresis (PFGE). They wrote about “similar, though not identical numbers and sizes of individual chDNAs” and “one can conclude that there is a close relatedness between x-1 and x-5” (Pinevich et al., 1997, p. 125). Thus, the number and size of chromosomes in two morphologically identical strains (x-1 and x-5 CALU) of *Amoeboaphelidium protococcarum* differ. These data show the occurrence of cryptic diversity and the importance of genetic studies of different strains within the same aphelid morphospecies. The genetic heterogeneity of morphologically indistinguishable strains is usual for some protists, e.g., syngene of ciliates (Lynn, 2008). However, further study may reveal the ultrastructural differences, which are still poorly known for aphelids.

## THE COMPARISON BETWEEN APHELIDS AND ROZELLIDS

*Rozella allomycis,* like the aphelids, also has endobiotic development and does not produce its own sporangium wall. The more important common characteristic is the ability of trophonts to phagocytose. This fact clearly separates the Aphelidea and Cryptomycota from the fungi and unambiguously supports the molecular phylogeny of both groups. The rozellids produce flagellated zoospores, and have zoosporic fungi and Oomycetes as their hosts, rather than algae, as do the aphelids (see details in Ecology of Aphelids in Comparison with *Rozella*).

The kinetid structure of the zoospores produced by *Rozella* is better known and differs essentially from that of *Aphelidium*. The flagellum in *Rozella* emerges from the bottom of a deep invagination at the cell’s posterior (Held, 1975). The flagellar kinetosome is long and has two prominent rhizoplasts connecting the kinetosome to the mitochondrion (Held, 1975). *Aphelidium* has a flagellum emerging from the flat surface of the zoospore and a relatively short kinetosome without the roots (Gromov and Mamkaeva, 1975). In *Rozella* the centriole lies at an angle of 45^o^ to the kinetosome (Held, 1975). Obvious peculiarities of kinetid in *Rozella* zoospores confirm its genetic difference from the aphelids, but the detailed reconstruction of *Aphelidium* kinetid is necessary.

*Rozella* zoospores have special endoplasmic reticulum (ER) cisterns closely associated to the nucleus (Held, 1975) reminding the inverted tube of *Pseudaphelidium*. No such cisterns or other traces of such tubes were found in *Aphelidium* (Gromov and Mamkaeva, 1975; Karpov, in preparation) and *Amoeboaphelidium* (Gromov and Mamkaeva, 1968, 1970b; Letcher et al., 2013). Thus, from the morphological perspective, the two latter genera appear to be at a greater distance from microsporidia than *Pseudaphelidium* and *Rozella.* At the same time, the *Aphelidium* might have retained ancestral features of the ARM branch, having a simpler life cycle with less complex uniflagellate zoospores.

The cyst wall of both *P. drebesii* and *Aphelidium chlorococcarum* stains with calcofluor white indicating the presence of chitin (Schweikert and Schnepf, 1997; Gromov, 2000). The cyst wall of *Rozella* is also composed of chitin (James and Berbee, 2012; James et al., 2013). The occurrence of chitin, and the presence of four chitin synthetase genes in *Rozella* contradicts former descriptions of Cryptomycota claiming that they have “cysts without a chitin/cellulose cell wall” (Jones et al., 2011b).

The presence of chitin cell walls and chitin synthetase genes in the whole ARM clade strongly suggests that the common ancestor of the fungi and ARM already possessed fungal-specific chitin biosynthesis.

## ECOLOGY OF APHELIDS IN COMPARISON WITH *ROZELLA*

All known species of *Rozella* and the aphelids are obligate parasitoids (biotrophs) and must be grown in culture with their hosts (Held, 1981; Gromov, 2000; Gleason et al., 2012). Therefore, their ecology cannot be disentangled from that of their hosts. One important ecological difference is that the hosts for *Rozella* species are zoosporic fungi and Oomycetes (heterotrophic stramenopiles), while aphelid genera have a wide variety of phytoplankton species as hosts. According to Held (1981) the hosts for the class Rozellidae are placed into four phyla of zoosporic true fungi and fungal-like organisms: Chytridiomycota, Blastocladiomycota, Monoblepharomycota, and Oomycota (Saprolegnialean and Peronosporalean galaxies). As previously stated, Held (1981) briefly speculated that some phytoplankton species might be *Rozella* hosts as well, but did not consider this possibility further. Other undescribed cryptomycotes attack diatom algae, as was shown by FISH (Jones et al., 2011a). According to Gromov (2000) the hosts for the class Aphelidae belong to the phyla Chlorophyta, Xanthophyta, and Bacillariophyta (**Table 1**). The most common hosts for the aphelids are found among the chlorococcous algae, and these host-parasite relationships are often genus specific (Fott, 1957, Gromov and Mamkaeva, 1966, 1968). A xanthophyte, *Tribonema gayanum* Pasher, is the most commonly reported host for aphelids (Gromov, 1972; Gromov et al., 2002), and this species is normally used to support the cultures of these parasitoids. In nature the aphelids prefer the eutrophic water basins, where they live on planktonic algae, epiphytic algae on aquatic plants, and soil surface near the temporary and permanent water reservoirs. Mamkaeva et al. (1974) showed that *Amoeboaphelidium protococcarum* regularly occurs, sometimes in high densities, in some ponds of the Kaliningrad region of Russia. In the Ribinskoye reservoir (Yaroslavl district, Russia) its density is relatively low, and has so far never been found in sphagnum bogs. At the same time this organism has been found in 6% of samples from water basins of the Soviet Union (Mamkaeva et al., 1974). The density of parasitoid population varies significantly in different water bodies. Gromov et al. (2002) investigated the distribution of *Aphelidium* and the very common chytridiomycete, *Rhizophydium*, in 10 stations in the Ladoga lake and adjacent water bodies in August 2000, and found *Aphelidium* in 6 and *Rhizophydium* in 10 stations. The number of infectious units (zoospores or infected algal cells) for *Aphelidium* varied from 0.01 to 0.92 per 300 ml of water, while for *Rhizophydium* it was from 0.1 to 1.6. This suggests that the infection level by *Aphelidium* is comparable with that of the most common freshwater representative of Chytridiomycota. Mohamed and Martiny (2011) investigated the fungal diversity along an estuarine salinity gradient in Rhode Island by sequencing a large set of environmental 18S rRNA genes. Aphelid plus rozellid sequences represented 9% of the 1095 “fungal” clones obtained, and around 25% of the phylotypes (28 out of 104). Aphelid and rozellid sequences were more abundant in freshwater marsh samples (12%), than in brackish (8%) or salt marshes (5%). The relative amount of aphelid and rozellid sequences and phylotypes was almost the same in the different libraries from Rhode Island marshes (Mohamed and Martiny, 2011), whereas normally there is a significant excess of rozellids over the aphelids in environmental samples.

**Table 1:** Strains and plasmids used in this study.

Parasitoid		Host	
Genus	Species	Genus and species	Phylum
*Aphelidium*	*deformans* Zopf, 1885	*Coleochaeta* sp.	Chlorophyta
	*melosirae* Scherffel, 1925	*Melosira varians*	Bacillariophyta
	*tribonemae* Scherffel, 1925	*Tribonema gayanum*, *Botridiopsis intercedens*	Xanthophyta
	*chlorococcarum* Fott, 1957	*Scenedesmus armatus,* other chlorococcous algae	Chlorophyta
	*chlorococcarum* f. *majus* Gromov et Mamkaeva, 1970	Chlorococcous algae	Chlorophyta
*Amoeboaphelidium*	*achnanthides* Scherffel, 1925	*Achnanthes* sp.	Bacillariophyta
	*protococcarum* Gromov et Mamkaeva, 1968. emend. Karpov	*Scenedesmus obliquus, Scenedesmus dimorphus, Scenedesmus minutum, Chlorococcum* sp., other chlorococcous algae	Chlorophyta
	*chlorellavorum* Gromov et Mamkaeva, 1968	*Chlorella* sp.	Chlorophyta
	*radiatum* Gromov et Mamkaeva, 1969	*Kirchniriella* sp., *Ankistrodesmus* sp.	Chlorophyta
*Pseudaphelidium*	*drebesii* Schweikert et Schnepf, 1996	*Thalassiosira punctigera,* other diatoms	Bacillariophyta

*Data were taken from Gromov (2000); Karpov et al. (2013), and Letcher et al. (2013).*

In the producer-based food webs *Rozella* species are secondary consumers while aphelids are primary consumers. It is likely that both *Rozella* and aphelids can play roles in regulating the size, composition and dynamics of populations of zoosporic true fungi and Oomycetes (heterotrophic stramenopiles) and phytoplankton. Species of* Rozella* and aphelids are common parasitoids and therefore are likely to be factors which determine ecosystem complexity, although quantitative data on host-parasitoid dynamics are not yet available. Also, it is likely that species of *Rozella* can regulate the population sizes of zoosporic true fungi and Oomycetes in detritus-based food webs. Without empirical data the impact of their roles in regulation of host populations remains unknown. Preliminary data suggest that some of these parasitoids are highly virulent, and that the cytoplasm of the host appears to be converted efficiently into the cytoplasm of the parasitoid (Held, 1981; Gromov, 2000).

There is evidence that virulence of different genotypes of parasitoids and sensitivity of different genotypes of hosts to infection are variable in host-parasitoid interactions in both the Cryptomycota and the Aphelidea. The host range for *R. allomycis* has been tested in the laboratory and appears to be very narrow, with only a few genotypes of *Allomyces* being susceptible to infection (Held, 1981). Gromov and Mamkaeva (1968) measured the susceptibility of 226 different strains of green and yellow-green algae to infection by four isolates of *Amoeboaphelidium* (x-1 through x-4 CALU). Some cultures were susceptible to infection by strains x-1, and x-4 (*Amoeboaphelidium protococcarum*), while others were resistant. The strain x-2 (*Amoeboaphelidium chlorellavorum*) infected only the *Chlorella* strains.

As previously stated, the motile propagules produced by species of *Rozella* and aphelids are different. All known *Rozella* species produce uniflagellate zoospores. Aphelids can produce either uniflagellate zoospores, amoebae without flagella or amoebae with flagella reduced in size (**Figure 2**). Zoospores are adapted for swimming in water while amoebae (even with a posterior immobile pseudocilium) are adapted to crawl on surfaces (Gleason and Lilje, 2009; Karpov et al., 2013). Interestingly, *Aphelidium melosirae* and *Aphelidium tribonemae* zoospores, despite having normal flagella, have been observed to crawl like amoebae (Gromov, 1972). They produce short lobopodia (*Aphelidium melosirae*), or filopodia (*Aphelidium tribonemae*), and crawl using pseudopodia while the immobile flagellum trails behind the zoospore.

**FIGURE 5:**
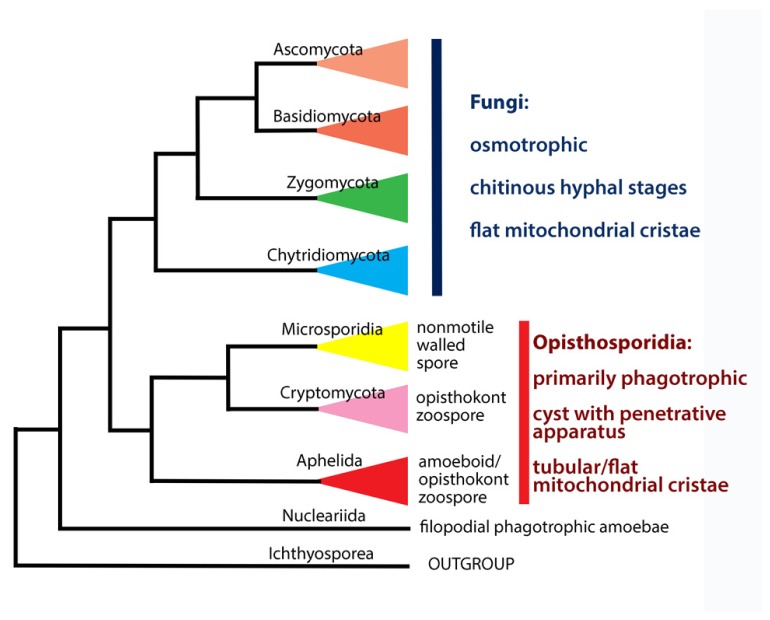
**Phylogenetic and taxonomic summary of early diverged groups of Holomycota**.

Thus, the aphelids retained the amoeboid nature in all three genera not only at trophic stage like *Rozella*, but also in propagules, which agrees with their more basal position in the molecular phylogenetic tree (**Figure 5**).

As previously stated both *Rozella* and the aphelid genera use the host cell wall as a zoosporangium. Therefore, these endobiotic species are likely to use less energy in reproduction than epibiotic species since they do not produce an extra structure for zoospore release. Since both groups have small thalli and most of the cytoplasm goes into the zoospores, reproduction is energy efficient. Therefore, a relatively high percentage of energy is likely to be transferred between trophic levels. Species in both groups are at the second or third trophic level as explained above so that bottom-up effects would impact on the entire food web above producers or primary consumers. Because of their small sizes (Held, 1981; Gromov, 2000) zoospores produced by *Rozella* and aphelids are likely to be easily eaten by filter feeding zooplankton and metazoans at the third or fourth trophic level (Gleason et al., 2008). At least one uncultured aphelid species has been detected in fecal pellets passed through the intestinal tract of detritus-feeding freshwater amphipod *Gammarus tigrinus* from Canada (Sridhar et al., 2011). Rozellids found in the feces of studied species of amphipods accounted for 21 out of the 74 “fungal” clones in the same analysis and clustered in 10 phylotypes (Sridhar et al., 2011). In this way the nutrients of the host plankton are recycled by the microbial loop while they remain in the euphotic zones.

## TAXONOMY

Presently, the Cryptomycota has a rank of phylum (Jones et al., 2011b), as has its sister group the Microsporidia in the ARM clade. But both phyla are sisters to the class Aphelidea (Karpov et al., 2013; **Figure 5**). Therefore, it is logical to change the class Aphelidea to a phylum – taxon of the same rank as Cryptomycota and Microsporidia. At the same time, the whole ARM branch is sister to all the Fungi *sensu lato,* and are not true Fungi. Thus, we now amend the taxonomy of the ARM-clade, and establish a new superphylum the Opisthosporidia with three phyla: Aphelida phyl. nov., Cryptomycota, and Microsporidia. This proposal is based on molecular phylogeny, morphological, ultrastructural and ecological characteristics of all three phyla discussed in this review.

### OPISTHOKONTA Cavalier-Smith, 1987

The Opisthokonta is divided into Holomycota and Holozoa, a division based solely on molecular characteristics. Cryptomycota and the whole ARM-clade (Opisthosporidia) are not true fungi (**Figure 5**), as has also been noted by Cavalier-Smith (2013). Thus, along with nucleariids, one more branch has appeared at the Holomycota/Holozoa border. Given that more and more deep branches are appearing at this border, what has until recently appeared to be a clear distinction may become unstable, particularly with the acquisition of molecular data for pompholyxophryids, other rotospherids, and yet-unsampled potential Opisthokonta.

### OPISTHOSPORIDIA KARPOV, ALEOSHIN ET MIKHAILOV SUPERPHYLUM nov.

Opisthokont intracellular parasites/parasitoids with amoeboid vegetative stage. Invasive spores/cysts with chitin cell wall and specialized apparatus for penetration into host cell (penetration tube; posterior vacuole). If present, zoospores with filopodia and/or one posteriorly directed whiplash flagellum (functional or rudimentary). Phagotrophic or osmotrophic.

#### Etymology

Named by word combination of Opisthokont and sporae, making reference to the specialized penetration apparatus of the spore (in Microsporidia) or cyst (in two other phyla) characteristic for all three phyla Aphelida, Cryptomycota, and Microsporidia.

Superphylum includes three phyla (**Figure 5**): Microsporidia, Cryptomycota and Aphelida phyl. nov.

The phylum Microsporidia is well known and has a good description (Issi and Voronin, 2007; Williams and Keeling, 2011; Vávra and Lukeš, 2013). Microsporidia are protistan parasites of animals (predominantly insects and crustaceans) and rarely infect protists. Microsporidia have been known since 1882 by Balbiani and at present they account for 1300 to 1500 species, distributed in 187 genera. They are obligate intracellular parasites with a relatively uniform life cycle: a germinating spore injects the spore contents (sporoplasm) into the host cell by means of an explosively evaginable “injection tube” (polar tube or polar filament). The sporoplasm grows into cells called meronts, which divide into daughter meronts. The trophic stage is extremely simplified: it has a reduced genome, reduced ribosomes, poorly developed internal membrane system, lacks canonical dictyosomes, lost peroxisomes, and mitochondria are reduced to mitosomes which are unable to produce ATP. Microsporidia have developed a unique capacity to get ATP directly from the host cell, and became “energy parasites.” The meronts progressively fill the cytoplasm of the host cell, and then produce the chitin cell wall on their surface becoming the sporonts and then sporoblasts. Each sporoblast matures into a complex infective spore equipped with an injection apparatus. The infective spore is a dispersal stage, which can survive in the environment.

It was considered, that the presence of the injection apparatus in the spore is an autapomorphic character that sharply delineates microsporidia (Vávra and Lukeš, 2013). But it can be suggested, that the rozellids (Williams and Keeling, 2011) and aphelids, in the frame of the same phylogenetic lineage, have retained the primitive injection apparatus, which is homologous to the injection apparatus of microsporidia.

The description of the phylum Cryptomycota Jones et Richards, 2011 needs to be modified. The following description: “Fungi unicellular, zoospores single-celled with a single microtubular flagellum, and cysts without a chitin/cellulose cell wall. Forming epibiotic associations” (Jones et al., 2011b) contains many inaccuracies and certainly does not correspond to the genus *Rozella*, which is the only described genus (with approximately 20 valid species) in this phylum.

(1) “Fungi unicellular” – they are not fungi – it is a group of Opisthokonta, sister to Fungi.
(2) “Microtubular flagellum” – not a good word combination, as non-microtubular flagellum is unknown.
(3) “Cysts without a chitin/cellulose cell wall” – it is now known that the cyst of *R. allomycis* has a chitin cell wall and that this species contains four chitin synthetase genes (James and Berbee, 2012; James et al., 2013).
(4) “Forming epibiotic associations” – what are these associations? *Rozella* has cyst attached to the host surface, but does not live there, so is not epibiotic, rather it develops as an endobiont.

We propose that further improvements in the description of the phylum are necessary.

### PHYLUM CRYPTOMYCOTA (Jones et Richards, 2011), EMEND. KARPOV ET ALEOSHIN

Opisthokont intracellular parasitoids, predominantly of true fungi, Oomycetes (heterotrophic stramenopiles), and diatom algae with endobiotic phagotrophic amoeboid vegetative stage. Invasive cyst with short or long infective tube of penetration apparatus. Zoospore with posterior functional flagellum. 

At present, the diversity of Cryptomycota, shown by environmental sequences, is really huge (Lara et al., 2010; Jones et al., 2011a,b; Mohamed and Martiny, 2011; James et al., 2013; Karpov et al., 2013). They are found in marine, brackish and fresh waters, infect not only the fungi, like *Rozella,* but also some algae (Jones et al., 2011a). Unfortunately, the lack of clear borders for this phylum leads to the overestimation of their diversity. In the 18S rRNA gene phylogenetic trees reconstructed in the absense of aphelids (their environmental sequences were identified as the aphelids in 2013 only) and microsporidia (because of too long branches for this gene), any sequences lying between true fungi and *Nuclearia* were deemed Cryptomycota. Even in the presence of aphelids and microsporidia in the tree, these three independent branches were called Cryptomycota (Letcher et al., 2013) instead of ARM-clade.

In any case, a really broad divergence of these protists, which might be even wider than in the aphelids, suggests that we cannot exclude their parasitism on algae, and, perhaps, their saprotrophic mode of life. For further clarification we need more studies on the real organisms, to complement the molecular work that is being done.

### PHYLUM APHELIDA KARPOV, ALEOSHIN ET MIKHAILOV PHYLUM nov.

Opisthokont intracellular parasitoids of algae with phagotrophic amoeboid vegetative stage. Invasive cyst with short infective tube of penetration apparatus. Zoospore with pseudopodia and/or posteriorly directed functional or rudimentary flagellum.

*Rozella* and aphelids are morphologically similar to each other. But the genetic distance between them is very large, what we can certainly say now having sequenced genomes of *R. allomycis* (James et al., 2013) and multigene data of *Amoeboaphelidium protococcarum* (unpublished). Moreover, each species is well nested within the two large groups defined by the environmental sequences (**Figure 1**).

### CLASS APHELIDEA GROMOV, 2000

Amoeboid endobiotic parasitoids of algae. Dispersal stages in the life cycle, zoospores or amoebae, attach to a new host cell and encyst. Amoeboid body penetrates into the host’s cell through a cyst stalk. The intracellular amoeba engulfs the contents of the host’s cell, forming food vacuoles which transport the food into the central digestive vacuole. An excretory body is formed in the digestive vacuole. The amoeboid trophont grows into a plasmodium, which totally replaces the cytoplasm of a host cell; the multinuclear plasmodium divides into uninuclear amoeboid cells or uniflagellated zoospores. No specialized sporangium cell wall is formed by the parasitoid around the sporangium. Some species form intracellular resting spores.

Order Aphelidida Gromov, 2000. Diagnosis coincides with that of the class.

New order with the same name Aphelidida, proposed by Cavalier-Smith (2013) is not valid.

Family Aphelididae Gromov, 2000. Diagnosis coincides with that of the class.

## REVIEWED DIAGNOSES OF APHELID GENERA AND SPECIES

### APHELIDIUM (Zopf, 1885) GROMOV, 2000

Parasitoids of various species of algae. Forms rounded to oval zoospores, able to produce pseudopodia, with one posteriorly directed whiplash acronematic flagellum and one or several lipid globules (refractile granules). Vegetative development as described for the class. Resting spores rounded to oval with a thick smooth cell wall, and without an excretory body which is ejected from the spore before spore wall synthesis.

Type species of the genus Aphelidium deformans Zopf, 1885.

*Aphelidium deformans* Zopf, 1885. Parasitoid of a green alga *Coleochaeta*. Infected host cell is deformed, becoming abnormally large (up to 10 times vs. normal) with thickened cell wall. Zoospores 2–3 μm in diameter. Resting spores rounded to oval, 12–30 μm in diameter, with a large lipid granule.

*Aphelidium melosirae* Scherffel, 1925. Parasitoid of the diatom alga *Melosira varians* Ag. Zoospores oval, 4 × 6 μm, with several refractive granules. Flagellum is about 10 μm long. Zoospores are slightly amoeboid, can produce short lobopodia and move like amoebae having an immotile flagellum. Resting spores 12–14 × 10 μm.

*Aphelidium tribonemae* Scherffel, 1925. Parasitoid of a yellow-green alga *Tribonema*. Zoospores 2–3 μm in diameter, flagellum is about 7 μm long with long (5 μm) acronema. Zoospores can produce filopodia and move like amoebae with an immotile flagellum. The development of *Aphelidium tribonemae* was observed in *Tribonema gayanum* Pasch. and *Botridiopsis intercedens* Visch. et Pasch.

*Aphelidium chlorococcarum* Fott, 1957. Parasitoid of chlorococcous algae. Zoospores 1.5–2.0 μm in diameter. Flagellum about 8 μm long. Resting spores ellipsoid, 7.0 × 5.0–6.5 μm. Parasitoid ultrastructure from mass culture of *Scenedesmus armatus* Chod. was investigated by Schnepf et al. (1971).

*Aphelidium chlorococcarum* forma *majus* Gromov et Mamkaeva, 1970. Zoospores 2.0–3.0 μm in diameter, flagellum about 14 μm long. The ultrastructure of zoospores and vegetative stages investigated by Gromov and Mamkaeva (1975).

*A. lacerans* Bruyne, 1890 and *A. chaetophorae* Scherffel, 1925 do not correspond to the diagnosis of the genus (Gromov, 2000).

### *AMOEBOAPHELIDIUM* (SCHERFFEL, 1925) GROMOV, 2000, EMEND. KARPOV

Parasitoids of various species of algae. Amoeboid zoospores, with or without posterior pseudocilium, forming flat hyaline pseudopodium with subfilopodia, or filopodia of different length. Vegetative development as described for the class. Resting spores rounded to oval, with a thick cell wall.

*Type species of the genus Amoeboaphelidium achnanthides Scherffel, 1925*.

*Amoeboaphelidium achnanthides* Scherffel, 1925. Parasitoid of the diatom *Achnanthes*, amoebae about 2 μm long.

*Amoeboaphelidium protococcarum* Gromov et Mamkaeva, 1968, emend. Karpov. Parasitoid of chlorococcous algae, strains differ by the possible hosts. Amoebae 2.0–4.0 μm in diameter with posterior pseudocilium 7 μm long. Resting spores oval, 4–6 × 5–7 μm.

Type strains: x-1, x-4 CALU (Gromov and Mamkaeva, 1968).

*Amoeboaphelidium chlorellavorum* Gromov et Mamkaeva, 1968. Parasitoid of some species of the green alga *Chlorella*. Amoebae about 1 μm in diameter, extracellular cysts without a discernible stalk.

Type strain: x-2 CALU.

*Amoeboaphelidium radiatum* Gromov et Mamkaeva, 1969. Parasitoid of the chloroccous algae *Kirchniriella* and *Ankistrodesmus*. Amoebae 1–3 μm in diameter with limited motility, have very thin and long filopodia (10–12 μm). Development on the surface of solid culture media not observed.

Type strain: x-3 CALU.

### *PSEUDAPHELIDIUM* SCHWEIKERT ET SCHNEPF, 1996

Zoospores colorless, lacking a noticeable refractive granule, with one posteriorly directed whiplash flagellum with an acroneme. The body of the parasitoid penetrates the host’s cell with a special infection tube everting from the cyst. Vegetative development as described for the class. Plasmodium divides into amoeboid cells, which encyst being released from sporangium. New opisthokont zoospores leave the cysts.

*Type species of the genus Pseudoaphelidium* drebesii Schweikert et Schnepf, 1996.

*P. drebesii* Schweikert et Schnepf, 1996. Zoospores 5 μm long and 3 μm wide, flagellum 15 μm long. By the end of the development plasmodium forms a hollow sphere. It divides into rounded cells, from which amoeboid cells with very limited motility are formed. Amoebae encyst (cyst diameter 4–6 μm). 1 or 2, more often 4 zoospores release from the cyst after excystment. Parasitoid of marine planktonic diatoms *Thalassiosira punctigera* (Castracane) found in Hasle from the North Sea.

## Conflict of Interest Statement

The authors declare that the research was conducted in the absence of any commercial or financial relationships that could be construed as a potential conflict of interest.
